# Assessment of Thyroid Hormone Status Among Patients With Chronic Kidney Disease in a Tertiary Care Center: A Cross-Sectional Study

**DOI:** 10.7759/cureus.99501

**Published:** 2025-12-17

**Authors:** Adeena Haja, Mohamed Rizwan B, Jiya Michael, Jenish Babu A, Alex M Varghese, Vijila Berlin S

**Affiliations:** 1 Department of General Medicine, PPK Hospital, Marthandam, IND; 2 Department of Physiology, Believers Church Medical College Hospital, Thiruvalla, IND; 3 Department of Nephrology, SRM Medical College Hospital and Research Centre, Kattankulathur, IND; 4 Department of Forensic Medicine, Believers Church Medical College Hospital, Thiruvalla, IND; 5 Department of Child Health Nursing, Global College of Nursing, Nattalam, IND

**Keywords:** chronic kidney disease, hypothyroidism, low t3 syndrome, thyroid dysfunction, thyroid-stimulating hormone

## Abstract

Background: Chronic kidney disease (CKD) is a progressive condition characterized by an irreversible decline in renal function. Thyroid dysfunction is frequently observed in individuals with CKD due to impaired hormone metabolism, altered peripheral conversion, and reduced renal clearance. These changes may influence disease progression and overall clinical outcomes, making thyroid evaluation clinically relevant in CKD populations.

Aim: To assess the level of thyroid dysfunction in individuals with chronic kidney disease and to correlate these findings with the different stages of CKD.

Methods: This 18-month descriptive cross-sectional study included 90 patients with CKD attending the Dialysis Unit and Medicine Outpatient Department at SMIMS, Kulasekharam. A detailed clinical evaluation and laboratory investigations were performed for all participants. Serum urea, creatinine, electrolytes, albumin, lipid profile, and thyroid function tests, including thyroid-stimulating hormone (TSH), free triiodothyronine (fT3), and free thyroxine (fT4), were measured using an electrochemiluminescence immunoassay (ECLIA). CKD staging was defined using KDIGO 2021 eGFR criteria. Statistical analysis included one-way ANOVA, chi-square test, Fisher’s exact test, and Student’s t-test as appropriate, with p < 0.05 considered statistically significant.

Results: Of the 90 participants, 24 (27%) had normal thyroid function, whereas 66 (73%) exhibited thyroid dysfunction. Among these, 35 (48%) had overt hypothyroidism, 17 (19%) had low T3 syndrome, and 14 (15.6%) had subclinical hypothyroidism; no cases of hyperthyroidism were identified. Thyroid abnormalities were more frequent in advanced CKD. Mean TSH levels increased significantly from Stage 3 (3.8 ± 1.2 µIU/mL) to Stage 5 (5.9 ± 1.8 µIU/mL) (p = 0.001), while mean fT4 and fT3 values declined progressively across stages (p = 0.003 and p < 0.001, respectively). Low HDL was common, affecting 66.67% of patients.

Conclusion: Thyroid dysfunction, particularly overt hypothyroidism, low T3 syndrome, and subclinical hypothyroidism, was highly prevalent among CKD patients and demonstrated a significant association with advancing CKD stages. These findings highlight the importance of routine thyroid evaluation in CKD to support early detection and improved clinical management.

## Introduction

Chronic kidney disease (CKD) is a degenerative, non-communicable illness defined by the continuous and irreversible deterioration of renal functioning [1]. This deterioration affects several physiological systems, including metabolic, endocrine, excretory, and synthetic pathways, ultimately leading to the accumulation of nitrogenous waste products, various metabolic disturbances, and clinical manifestations [2]. Among the many systemic effects of CKD, alterations in thyroid hormone levels are particularly significant yet remain less well understood [3]. Thyroid dysfunction frequently coexists with renal impairment, potentially complicating disease progression and patient outcomes [4]. The pituitary gland releases thyroid-stimulating hormone (TSH), which regulates the production of thyroid hormones that impact glomerular filtration rate (GFR), renal blood flow, tubular reabsorption and secretion, and electrolyte homeostasis [5].

Chronic renal disease also has a major influence on the hypothalamic-pituitary-thyroid (HPT) axis. In CKD, progressive accumulation of uremic toxins and metabolic waste products disrupts multiple levels of the hypothalamic-pituitary-thyroid (HPT) axis [6]. Uremia interferes with hypothalamic secretion of TRH, leading to blunted stimulation of pituitary TSH release. Additionally, metabolic toxins impair TSH glycosylation and bioactivity, resulting in the secretion of TSH molecules that are immunoreactive but functionally less active [7]. This contributes to altered feedback regulation across CKD stages. With advancing CKD (Stages 3-5), decreased renal clearance of iodine leads to iodine retention, which suppresses thyroid hormone synthesis via the Wolff-Chaikoff effect. Uremic inhibition of deiodinase enzymes further reduces peripheral conversion of T4 to T3, resulting in progressively lower fT3 levels, especially in Stages 4 and 5 [8]. Protein-binding abnormalities caused by metabolic acidosis and the presence of competitive binding toxins decrease the availability of free thyroid hormones [9].

These mechanisms correspond with the stage-wise biochemical trends observed in the present study: rising TSH levels (from 3.8 μIU/mL in Stage 3 to 5.9 μIU/mL in Stage 5), a gradual decline in fT4, and the most pronounced reduction in fT3 (p < 0.001) [6]. Thus, worsening uremia and toxin accumulation across CKD stages directly contribute to impaired thyroid synthesis, secretion, and peripheral hormone metabolism [9]. In addition, the presence of uremic toxins and competitive binding inhibitors can alter the protein binding of circulating thyroxine [10]. As GFR declines, iodine clearance decreases, leading to its accumulation, which can inhibit thyroid hormone synthesis through the Wolff-Chaikoff effect [11].

Recent research has focused on understanding how systemic inflammation in CKD further affects thyroid function. Chronic kidney disease is characterized by a persistent state of low-grade inflammation driven by elevated cytokines such as interleukin-6 (IL-6), tumor necrosis factor-α (TNF-α), and C-reactive protein (CRP) [12]. These inflammatory mediators suppress hypothalamic secretion of TRH and alter pituitary responsiveness, resulting in impaired TSH pulsatility and decreased biological activity of secreted TSH. Inflammation also inhibits type 1 and type 2 deiodinase enzymes, which are essential for the peripheral conversion of T4 to the biologically active [13] T3. As a result, patients, especially those in advanced CKD stages, exhibit reduced fT3 levels, a pattern characteristic of nonthyroidal illness syndrome (NTIS), also known as sick euthyroid syndrome.

Additionally, pro-inflammatory cytokines increase the expression of type 3 deiodinase, an enzyme that inactivates thyroid hormones by converting T4 to reverse T3 (rT3). This shift promotes further decline in active T3 concentrations. Inflammatory molecules also modify the thyroid hormone-binding proteins and reduce albumin levels, decreasing the transport and bioavailability of thyroid hormones [14]. Collectively, this chronic inflammatory state contributes to the progressive pattern observed in our study: declining fT3 and fT4, along with rising TSH across CKD stages, reflecting both central and peripheral disruptions of thyroid homeostasis. These mechanisms also explain why individuals with advanced CKD may demonstrate biochemical profiles consistent with nonthyroidal illness syndrome, a state in which thyroid hormone alterations arise secondary to chronic systemic disease rather than intrinsic thyroid pathology. Given the elevated mortality and high prevalence of cardiovascular disease among patients with chronic kidney disease, recognizing thyroid dysfunction in this population is clinically important, as it may contribute to worsening metabolic disturbances and adverse outcomes [15].

Different types of thyroid dysfunction exert varying clinical effects on CKD progression. Overt hypothyroidism has been shown to accelerate renal functional decline by reducing renal blood flow, impairing glomerular filtration, and worsening fluid and electrolyte imbalances [2]. In the present study, overt hypothyroidism was the most common abnormality (48%), and its higher prevalence in Stage 4-5 CKD suggests a strong association with advancing renal impairment, as supported by earlier findings [4]. Subclinical hypothyroidism, though milder, may still contribute to CKD progression by increasing systemic vascular resistance and impairing cardiovascular function, contributing to altered renal hemodynamics [9]. Its presence exclusively in Stage 5 CKD in our data emphasizes its relevance in advanced renal disease.

Low T3 syndrome, identified in 19% of patients, is widely considered a marker of non-thyroidal illness in CKD [3]. Reduced T3 levels correlate strongly with declining eGFR and increased inflammatory burden, which explains their association with disease severity [6]. In our study, low T3 syndrome demonstrated the strongest correlation with worsening CKD stages, particularly Stages 4 and 5, indicating metabolic stress and reduced renal reserve. Hyperthyroidism, though absent in our study population, is known to increase renal perfusion and GFR transiently, yet may worsen metabolic imbalance in CKD [1]. Even though no cases were observed, existing evidence suggests that uncontrolled hyperthyroidism can adversely affect CKD outcomes [5]. Overall, the spectrum of thyroid dysfunction, especially overt hypothyroidism and low T3 syndrome, appears to contribute significantly to clinical deterioration and may influence the pace of CKD progression [8].

Objectives of the study

The objective of this study is to determine the prevalence and patterns of thyroid dysfunction in individuals with chronic kidney disease and to evaluate the association between thyroid hormone abnormalities and CKD stages.

## Materials and methods

Study design

Participants in this cross-sectional descriptive study were chronic kidney disease (CKD) patients enrolled at “Dialysis Unit and Medicine Outpatient Department, Sree Mookambika Institute of Medical Sciences (SMIMS), Kulasekharam, Kanyakumari District, Tamil Nadu.” It was conducted for a total of 18 months, from October 2022 to April 2024. The study received ethical clearance from SMIMS's Institutional Human Ethics Committee (IHEC), with approval reference number SMIMS/IHEC No: 1/Protocol No: 17/2022.

Study population

Patients between the ages of 18 and 70 who had been diagnosed with chronic kidney disease were included in the study. A consecutive sampling procedure was employed, in which every patient who attended the Dialysis Unit and the Medicine Outpatient Department of SMIMS during the 18-month study period was screened for eligibility. All patients who met the inclusion criteria and provided informed consent were enrolled sequentially until the required sample size was reached. Individuals were excluded if they had conditions other than CKD that could transiently alter thyroid function, such as recent surgery, active infections, acute inflammatory conditions, burns, trauma, or liver disease, or if they had a known history of thyroid disorders or were taking medications known to interfere with thyroid function (including amiodarone, steroids, beta-blockers, dopamine, phenytoin, iodine-based preparations, or estrogen therapy).

Sample size

Previous studies showing a 53% incidence of thyroid dysfunction in patients with chronic kidney disease were used as the expected prevalence (P) for determining the sample size [11]. This prevalence aligns with the demographic characteristics of the CKD population typically seen in our region, where most individuals are middle-aged or older adults living with long-standing hypertension, diabetes, and progressive renal impairment. Patients attending the Dialysis Unit and Medicine Outpatient Department at SMIMS predominantly fall within the 40- to 70-year age group, with a higher proportion of males and many presenting with advanced CKD stages. These demographic patterns support the use of the 53% prevalence estimate for accurate sample size determination.

The required sample size for this cross-sectional study was calculated using the standard formula for estimating a proportion:

$n=\left(Z^2 \times P \times(1-P)\right) / d^2$

where Z = 1.96 for a 95 percent confidence level, P = 0.53 (expected prevalence), d = 0.10 (allowable margin of error).

Substituting these values:

n = (1.96² × 0.53 × 0.47) / 0.10² n = 86

Thus, the minimum required sample size was 86 participants. To enhance the reliability, precision, and statistical power of the study, a total of 90 eligible individuals were ultimately included.

Data collection was carried out by a team of trained medical personnel consisting of postgraduate residents from the Department of General Medicine and nursing staff assigned to the Dialysis Unit and Medicine Outpatient Department. Before the start of the study, all investigators underwent structured training conducted by the principal investigator. This training covered patient interviewing techniques, standardized procedures for obtaining clinical history, proper methods for conducting physical examinations, uniform documentation practices, and protocols for sample collection and handling to ensure consistency and reliability across all observations.

Each participant underwent a detailed clinical history assessment followed by a comprehensive physical examination. Laboratory investigations included routine urine microscopy, complete blood count, hemoglobin estimation, peripheral smear evaluation, and measurement of red cell indices such as mean corpuscular volume (MCV) and mean corpuscular hemoglobin (MCH). These tests were performed as part of the standard clinical assessment for patients with chronic kidney disease to evaluate anemia, nutritional status, and baseline hematological abnormalities commonly associated with CKD. In addition to these routine investigations, thyroid function tests, including TSH, free T3, and free T4, were carried out to assess thyroid status as required by the study objectives. All laboratory tests were selected based on their clinical relevance to CKD and the need to correlate renal function with thyroid hormone alterations.

The levels of serum creatinine and blood urea were measured to evaluate renal function and to calculate the estimated glomerular filtration rate (eGFR). CKD stages were defined according to the KDIGO 2021 classification, where Stage 3 corresponds to an eGFR of 30-59 mL/min/1.73 m², Stage 4 to 15-29 mL/min/1.73 m², and Stage 5 to less than 15 mL/min/1.73 m². The CKD-EPI 2021 creatinine equation was utilized [1]. Additional biochemical analyses included total protein, calcium, phosphorus, serum lipid profile, and serum albumin. Echocardiography (ECHO) was also performed to evaluate left ventricular function as part of the cardiac assessment [6].

Thyroid function assessment

For the quantitative evaluation of thyroid parameters, such as thyroid-stimulating hormone (TSH), free triiodothyronine (free T3), free thyroxine (free T4), and total triiodothyronine (total T3), venous blood samples were collected in non-heparinized serum bottles. The samples were analyzed using the electrochemiluminescence immunoassay method [4]. The reference ranges applied were 0.34-5.2 μIU/ml for TSH, 2.5-3.9 ng/ml for free T3, and 0.6-1.1 ng/dl for free T4.

Based on these values, thyroid dysfunction was classified into four groups: low T3 syndrome (reduced free T3 levels with normal free T4 and TSH), hyperthyroidism (elevated free T3 and free T4 with suppressed TSH), hypothyroidism (raised TSH with reduced free T3 and free T4), and subclinical hypothyroidism (normal free T3 and free T4 with elevated TSH) [3]. Low T3 syndrome is widely regarded as a biochemical expression of nonthyroidal illness syndrome, which is common in chronic systemic diseases such as CKD.

Statistical analysis

Before statistical analysis, all gathered data were consolidated and summarized. Frequencies and percentages were used to describe categorical data, whereas the mean ± standard deviation was used for continuous variables. Fisher’s exact test or the chi-square test, depending on data distribution, was applied to examine relationships between categorical variables. For comparisons of continuous variables, the Student’s t-test was used when data were normally distributed, and the Mann-Whitney U test was used for non-normally distributed data [16].

One-way ANOVA was additionally employed to compare mean thyroid function parameters, including thyroid-stimulating hormone, free triiodothyronine, and free thyroxine, across the different stages of chronic kidney disease. A p-value less than 0.05 was considered statistically significant for all analyses.

## Results

Demographic profile of the study population

Participants of this research were between the ages of 18 and 70. More than half of the participants were between the ages of 46 and 65. The participants' average age was 54.7 ± 12.3, and the ratio of male to female was 1.4:1. With 72 (80%) male participants, the study's male group was larger than its female group. As shown in Table 1, the mean TSH levels increased progressively across CKD stages, accompanied by corresponding declines in fT3 and fT4 values. The reduction in fT3 was particularly notable, consistent with the biochemical pattern typically seen in nonthyroidal illness (low T3 state).

**Table 1:** Baseline characteristics of the study population (n = 90) eGFR: estimated glomerular filtration rate, CKD: chronic kidney disease, LDL: low-density lipoprotein, VLDL: very-low-density lipoprotein, HDL: high-density lipoprotein, fT3: free triiodothyronine, fT4: free thyroxine, TSH: thyroid-stimulating hormone

Characteristic	Value
Age (years)	54.7 ± 12.3 (range 18–80)
Age distribution	46–65 years: >50% of participants
Gender	Male: 72 (80%); Female: 18 (20%)
Male-to-female ratio	1.4: 1
CKD Stage	Stage 3: 27 (30.2%); Stage 4: 32 (37.2%); Stage 5: 26 (30.2%)
Mean eGFR (mL/min/1.73 m²)	19.4 ± 8.2
Hemoglobin (g/dL)	9.6 ± 1.4
Anemia prevalence	80 (93.1%)
Peripheral smear	Normocytic normochromic: 67 (77.9%); Dimorphic: 7 (8.1%); Microcytic hypochromic: 12 (14.0%)
Blood urea (mg/dL)	108.6 ± 32.4
Serum creatinine (mg/dL)	6.2 ± 2.1
Serum albumin (g/dL)	3.4 ± 0.5
Total protein (g/dL)	6.5 ± 0.7
Calcium (mg/dL)	8.1 ± 0.6
Phosphorus (mg/dL)	5.3 ± 0.9
Dyslipidemia	Present in 49 (57%)
Lipid abnormalities	High LDL/VLDL: 23%; High triglycerides: 16.67%; Total cholesterol >200 mg/dL: 4%; Low HDL: 66.67%
Thyroid function status	Normal: 24 (27%); Abnormal: 66 (73%)
Types of thyroid dysfunction	Low fT3: 45.56%; Low fT4: 30%; High TSH: 55%

Distribution of CKD stages and renal function parameters

Among the study population, 27 (30.2%) of the patients had Stage 3 CKD, 32 (37.2%) had Stage 4 CKD, and 26 (30.2%) had Stage 5 CKD according to eGFR determined using the CKD-EPI 2021 creatinine formula. Average eGFR was 19.4 ± 8.2 ml/min/1.73 m².

Hematological and biochemical findings

The hemoglobin level was 9.6 ± 1.4 g/dl on average, and anemia was seen in 80 individuals (93.1%). Peripheral smear findings showed that 67 patients (77.9%) had normocytic normochromic anemia, seven patients (8.1%) had a dimorphic appearance, and 12 patients (14.0%) exhibited microcytic hypochromic anemia.

The average blood urea level was 108.6 ± 32.4 mg/dL, and the mean serum creatinine level among the participants was 6.2 ± 2.1 mg/dL. While overall protein was 6.5 ± 0.7 g/dl, calcium was 8.1 ± 0.6 mg/dl, and phosphorus was 5.3 ± 0.9 mg/dl, the serum albumin level was 3.4 ± 0.5 g/dl.

Lipid profile and dyslipidemia

A total of 49 patients (57.0%) had dyslipidemia. Elevated total cholesterol above 200 mg/dL was observed in 4% of patients, while high triglyceride levels above 160 mg/dL were present in 16.67%. Elevated LDL levels were found in 23%, and reduced HDL levels were noted in 66.67%.

Thyroid function status

**Figure 1:**
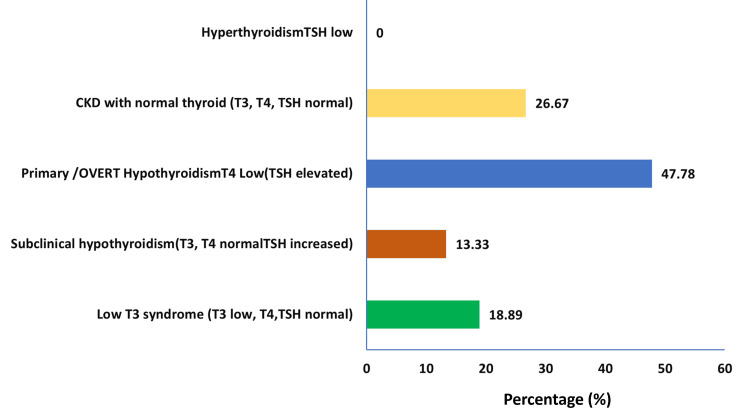
Descriptive analysis of thyroid abnormality Created by authors

Of the 90 people in the study, 24 (27%) had normal thyroid function at the time of the investigation, whereas 66 (73%) had abnormal thyroid function. TSH levels were within the normal range in 45% of patients and elevated in 55%. Low fT3 levels were observed in 45.56% of participants, and low fT4 levels were noted in 30%. The reference range for fT3 in this study was 2.5-3.9 pg/mL, and for fT4 it was 0.6-1.1 ng/dL. TSH levels are between 1.1 and 5.2. The distribution of thyroid function abnormalities among CKD patients is given in Figure 1.

Correlation of thyroid function with CKD stages

The mean serum TSH levels showed a progressive increase across the CKD stages, rising from 3.8 ± 1.2 µIU/mL in Stage 3 to 5.9 ± 1.8 µIU/mL in Stage 5, and this trend was confirmed using one-way ANOVA, which demonstrated a statistically significant difference between the stages (p = 0.001). Likewise, the mean free T4 values demonstrated a gradual decline from 0.94 ± 0.14 ng/dL in Stage 3 to 0.72 ± 0.15 ng/dL in Stage 5, with statistical significance (p = 0.003). Similarly, mean free T3 levels decreased markedly from 3.05 ± 0.38 pg/mL in Stage 3 to 2.21 ± 0.29 pg/mL in Stage 5, showing the most pronounced reduction among the three parameters (p < 0.001).

These findings collectively demonstrate a significant deterioration of thyroid function with declining renal function, as defined by the KDIGO 2021 eGFR-based CKD staging criteria [17]. Table 2 summarizes the progressive alteration of thyroid parameters across the different stages of CKD.

**Table 2:** Correlation of TFT values with CKD stage Source: [17] CKD: Chronic kidney disease, TFT: Thyroid function test

CKD Stage	eGFR (mL/min/1.73 m²)	Description	TSH (µIU/mL) Mean ± SD	fT4 (ng/dL) Mean ± SD	fT3 (pg/mL) Mean ± SD
Stage 3	30 – 59	Moderate decrease in eGFR	3.8 ± 1.2	0.94 ± 0.14	3.05 ± 0.38
Stage 4	15 – 29	Severe decrease in eGFR	4.6 ± 1.4	0.88 ± 0.16	2.78 ± 0.34
Stage 5	< 15 (for dialysis)	Kidney failure	5.9 ± 1.8	0.72 ± 0.15	2.21 ± 0.29
p value	—	—	0.001	0.003	< 0.001

Correlation between thyroid disorders and CKD stages

Low T3 syndrome showed the strongest and most significant correlation with advanced CKD, especially at Stage 4 (r=0.39, p=0.02) and Stage 5 (r=0.32, p=0.001). Subclinical hypothyroidism was identified exclusively in Stage 5 CKD, exhibiting a weak yet significant positive correlation, whereas overt hypothyroidism displayed a weak-to-moderate link across Stages 3-5, with increased prevalence in later stages. No significant link was observed between normal thyroid function and Stage 5 CKD; however, a substantial correlation was identified in Stage 4, potentially because of the smaller sample size. The correlation between thyroid disorders and stage 5 CKD is presented in Table 3.

**Table 3:** Correlation between stage 5 renal failure and thyroid disorders Source: [17] eGFR: estimated glomerular filtration rate

eGFR	Thyroid disease	Number	r value	p-value
Stage 5 eGFR<15	Low T3 syndrome	14	0.32	0.001
	Subclinical hypothyroidism	14	0.16	0.02
	Hypothyroidism	30	-0.12	0.004
	Normal	19	0.043	0.38

The correlation between thyroid disorders and stage 4 CKD is shown in Table 4, highlighting a moderate association of hypothyroidism and low T3 syndrome with declining renal function.

**Table 4:** Correlation between stage 4 renal failure and thyroid disorders Source: [17] eGFR: estimated glomerular filtration rate

eGFR	Thyroid disease	Number	r value	p-value
Stage 4 eGFR<15-29	Low T3 syndrome	3	0.39	0.02
	Subclinical hypothyroidism	0	-	-
	Hypothyroidism	4	0.26	0.001
	Normal	3	0.43	0.02

The relationship between thyroid function and stage 3 CKD is detailed in Table 5, indicating a mild correlation of hypothyroidism with early renal impairment.

**Table 5:** Correlation between stage 3 renal failure and thyroid disorders Source: [17]

eGFR	Thyroid disease	Number	r value	p-value
Stage 3 eGFR<30-59	Low T3 syndrome	0	-	-
	Subclinical hypothyroidism	0	-	-
	Hypothyroidism	2	0.16	0.01
	Normal	1	0.33	0.1

## Discussion

The primary goal of the current study was to identify thyroid functioning abnormalities in individuals with chronic kidney disorders. There have been prior reports of aberrant thyroid function in these individuals. Nevertheless, in most instances, it remains undiagnosed because of challenges in carrying out investigations or interpreting laboratory findings. Previous research has reported variable results. Since thyroid dysfunction has been shown to accelerate the progression of chronic renal disease and increase morbidity and mortality, thyroid hormone evaluation should be incorporated into the biochemical assessment of affected individuals [3]. Symptoms and laboratory abnormalities often do not correlate, underscoring the well-established need to integrate clinical assessment with biochemical evaluation when interpreting thyroid function in patients with chronic illnesses such as CKD. In this study, thyroid function assays were performed to identify abnormalities and correlate them with CKD stages. A total of 66 (73%) patients demonstrated thyroid hormone disturbances, while 24 (27%) had normal thyroid function. Comparable studies have reported thyroid dysfunction in 53% [11] and 40% [12] of CKD patients.

Participants ranged in age from 18 to 80 years, with more than 67% between 36 and 65 years, consistent with earlier studies reporting similar age distribution [13]. The study population had a predominance of males (80%), among whom thyroid abnormalities were more frequently identified. These findings are comparable to previous research demonstrating male predominance in CKD with thyroid dysfunction [11]. A significant association between lipid abnormalities and CKD was observed in the form of reduced HDL and elevated LDL, VLDL, triglycerides, and total cholesterol. Similar trends have been reported, with CKD patients, especially those receiving hemodialysis, exhibiting lower HDL and markedly increased triglycerides and VLDL [14]. Other studies have shown that non-dialysis CKD patients also exhibit lipid derangements such as elevated triglycerides, reduced HDL, reduced LDL, and normal or low total cholesterol [15].

Thyroid hormone abnormalities in CKD are well documented, including significant reductions in total triiodothyronine and thyroxine (p < 0.0001) and a corresponding rise in TSH (p = 0.0002) [16]. Similar to the present findings, prior studies have noted considerably higher serum urea and creatinine levels compared to controls. Other reports have shown an 11.7% prevalence of thyroid dysfunction, with subclinical hypothyroidism forming the majority [17], and characteristic lipid and hematologic alterations associated with hypo- and hyperthyroid states in CKD patients. In the present study, thyroid abnormalities were most frequent in stage 5 CKD, followed by stage 4, with fewer abnormalities in earlier stages. This distribution is in line with previous studies reporting 52% of patients in stage V and 22% in stage IV, with progressive biochemical decline as renal function worsens [11,18]. In the current study population, 13.33% had subclinical hypothyroidism, 48% demonstrated reduced thyroid hormone levels, and 19% presented with low T3 syndrome. No laboratory pattern suggestive of hyperthyroidism was identified. Comparable studies have documented overt hypothyroidism in 4.8% and subclinical hypothyroidism in 29% of patients, with the majority in stage 5 CKD [19].

Age, disease severity, and hemodialysis status were strongly associated with thyroid dysfunction, as shown in prior studies [20]. Low T3 syndrome was absent in stages II and III in this study, but present in 17% of the sample, predominantly in stage 5 CKD. Earlier research similarly reports low T3 clustering among advanced CKD cases [21,22], reinforcing the strong link between disease progression and altered thyroid biochemistry. The present findings also align with reports showing increasing prevalence of thyroid dysfunction with CKD severity [11], including stage-wise increases in subclinical hypothyroidism and progressive reductions in T3/T4, accompanied by rising TSH levels (p = 0.04). These biochemical trends reflect impaired renal handling of iodine, altered hormone metabolism, and systemic illness-related changes that intensify with declining kidney function.

A key observation in this study is that the pattern of thyroid dysfunction, particularly the marked reduction in triiodothyronine and thyroxine concentrations in stage 5 CKD, accompanied by only modest elevation in TSH, closely resembles sick euthyroid syndrome, also termed nonthyroidal illness syndrome (NTIS). NTIS is characterized by decreased circulating thyroid hormones in the absence of intrinsic thyroid pathology and is frequently observed in individuals with chronic systemic disease. Mechanisms such as systemic inflammation, metabolic stress, malnutrition, uremic toxin accumulation, altered protein binding, and impaired conversion of T4 to T3 in advanced CKD contribute to this hormonal pattern. The predominance of older individuals and stage 5 CKD patients in this study likely enhanced the expression of NTIS-like biochemical profiles.

Importantly, this hormonal pattern differs from primary hypothyroidism, which typically presents with a markedly elevated TSH. The modest TSH rise observed in the present study supports the interpretation that many abnormalities reflect NTIS rather than true thyroid gland failure. Recognizing NTIS in CKD is clinically essential because it represents an adaptive physiological response rather than a condition that routinely requires thyroid hormone replacement therapy. Failure to differentiate between NTIS and primary hypothyroidism may lead to misinterpretation of results and unnecessary treatment. The progressive decline in free T3 and free T4 observed with worsening CKD severity in this study further reinforces the characterization of these abnormalities as NTIS, a documented phenomenon in advanced chronic illnesses.

Strengths and limitations

Strengths of this study include the use of standardized laboratory methods for thyroid function assessment, clear CKD staging based on KDIGO guidelines, and detailed stage-wise biochemical analysis. However, certain limitations must be acknowledged. This was a single-center study with a relatively modest sample size, which may limit generalizability. The cross-sectional design precludes establishing causality, and some clinical variables, such as nutritional status and inflammatory markers, were not assessed. These factors should be considered while interpreting the findings.

## Conclusions

The present study demonstrates a clear association between chronic kidney disease (CKD) severity and alterations in thyroid function, with many patients exhibiting biochemical patterns within the hypothyroid range. However, in advanced CKD, these abnormalities often reflect the physiological adaptations of nonthyroidal illness syndrome (NTIS), also known as sick euthyroid syndrome, rather than true primary hypothyroidism. A stage-wise decline in free triiodothyronine and free thyroxine, accompanied by only modest elevations in thyroid-stimulating hormone, was observed, an endocrine profile characteristic of NTIS. Low T3 syndrome and subclinical hypothyroidism were also noted, whereas no cases of hyperthyroidism were identified. These hormonal alterations may arise from uremic toxin accumulation, impaired iodine handling, systemic inflammation, and reduced peripheral conversion of T4 to T3, mechanisms well documented in chronic systemic illness. The predominance of NTIS-like findings among Stage 5 CKD patients underscores the importance of distinguishing NTIS from primary hypothyroidism, as NTIS does not routinely require thyroid hormone replacement therapy. Routine assessment and careful interpretation of thyroid function in CKD are therefore essential to avoid unnecessary treatment, support accurate diagnosis, and optimize clinical management. Recognizing whether thyroid abnormalities reflect primary dysfunction or NTIS is crucial for improving patient outcomes, particularly in advanced CKD.
